# Molecular-docking study of malaria drug target enzyme transketolase in *Plasmodium falciparum* 3D7 portends the novel approach to its treatment

**DOI:** 10.1186/s13029-015-0037-3

**Published:** 2015-05-22

**Authors:** Md. Anayet Hasan, Md. Habibul Hasan Mazumder, Afrin Sultana Chowdhury, Amit Datta, Md. Arif Khan

**Affiliations:** Department of Genetic Engineering and Biotechnology, Faculty of Biological Sciences, University of Chittagong, Chittagong, 4331 Bangladesh; Department of Biotechnology and Genetic Engineering, Mawlana Bhashani Science and Technology University, Santosh, Tangail, 1902 Bangladesh

**Keywords:** Transketolase, *Plasmodium falciparum* 3D7, Homology modeling, Drug target, Docking studies

## Abstract

**Background:**

Malaria has been a major life threatening mosquito borne disease from long since. Unavailability of any effective vaccine and recent emergence of multi drug resistant strains of malaria pathogen *Plasmodium falciparum* continues to cause persistent deaths in the tropical and sub-tropical region. As a result, demands for new targets for more effective anti-malarial drugs are escalating. Transketolase is an enzyme of the pentose phosphate pathway; a novel pathway which is involved in energy generation and nucleic acid synthesis. Moreover, significant difference in homology between *Plasmodium falciparum* transketolase (Pftk) and human (*Homo sapiens*) transketolase makes it a suitable candidate for drug therapy. Our present study is aimed to predict the 3D structure of *Plasmodium falciparum* transketolase and design an inhibitor against it.

**Results:**

The primary and secondary structural features of the protein is calculated by ProtParam and SOPMA respectively which revealed the protein is composed of 43.3 % alpha helix and 33.04 % random coils along with 15.62 % extended strands, 8.04 % beta turns. The three dimensional structure of the transketolase is constructed using homology modeling tool MODELLAR utilizing several available transketolase structures as templates. The structure is then subjected to deep optimization and validated by structure validation tools PROCHECK, VERIFY 3D, ERRAT, QMEAN. The predicted model scored 0.74 for global model reliability in PROCHECK analysis, which ensures the quality of the model. According to VERIFY 3D the predicted model scored 0.77 which determines good environmental profile along with ERRAT score of 78.313 which is below 95 % rejection limit. Protein-protein and residue–residue interaction networks are generated by STRING and RING server respectively. CASTp server was used to analyze active sites and His 109, Asn 108 and His 515 are found to be more positive site to dock the substrate, in addition molecular docking simulation with Autodock vina determined the estimated free energy of molecular binding was of −6.6 kcal/mol for most favorable binding of 6′-Methyl-Thiamin Diphosphate.

**Conclusion:**

This predicted structure of Pftk will serve first hand in the future development of effective Pftk inhibitors with potential anti-malarial activity. However, this is a preliminary study of designing an inhibitor against *Plasmodium falciparum* 3D7; the results await justification by *in vitro* and *in vivo* experimentations.

## Background

The genus *Plasmodium* is responsible pathogen for malarial infection in human and other mammalian species [1]. This disease exists in most of the tropical and subtropical regions including Asia, America and Sub-Saharan Africa. Though there are four species (*Plasmodium falciparum, Plasmodium vivax, Plasmodium ovale,* and *Plasmodium malariae*) have been detected from the *Plasmodium* genus for causing the disease, the most responsible and virulent among them is *Plasmodium falciparum* [2–5]. It has a wide host range and is responsible for causing the severe form of malaria. Malaria is transmitted in humans by the Anopheles mosquito. The infected Anopheles mosquito acts as a vector and harbors the *Plasmodium* [6]. Infected individual may suffer from fever, neurological symptoms, opisthotonous, seizures and even can progress to coma or death. According to World Health Organization (WHO) about 1.2 million people were killed in 2010 due to malaria and another 219 million cases of this disease were documented [7].

Recent rise in the death rate due to malaria is concerning alarmingly as traditional treatment is becoming obsolete. High price and problems related with distribution of drug to malaria affected poor communities (endemic areas) especially in Sub-Saharan Africa made the situation worse. Considering the scientific ground eradication of malaria is supposed to be a complex one. Cases of anti-malarial drug resistance have been growing expotentially as well as more cases are being recorded with *P. falciparum* strain’s drug-resistance that is accounted for about 60 percent of death [8–11]. Another challenge with malarial extermination is that a single-cell parasite is good enough for causing it as, it has the ability to escape human immune system. Even if a patient recovers and contracts from malaria, there is no guarantee that he or she will not be infected by malaria in future. These complications make it difficult to establish a proven vaccine for malaria. In case of other viral disease like measles, vaccine that carries a weakened strain of the virus has been injected into the blood stream which allows the body to create immunity to that virus in future infection. With malaria parasite, human body cannot develop this type of immunity as the malaria parasite go thorough modifications continuously [12]. Considering all these reasons, it is crucial to find out a new tool that would allow the scientist community to stay one step ahead of more affordable drugs and practical formulations.

With the completion of the genome sequencing of *P. falciparum*, it has been revealed that working with specific metabolic pathway of the parasite could pave a way for new mode of action against it. In *P. falciparum* one of the most fundamental metabolic pathways is the pentose phosphate pathway (PPP) which has been reported to play active role in *P. falciparum* infected erythrocytes [13, 14]. It can generate reducing equivalents in the form of NADPH. This pathway has an oxidative and a non-oxidative arm where the non-oxidative arm is operated by an enzyme, named transketolase. Transketolase serves different roles in malarial parasite including pentose sugar supply for nucleotide synthesis, helps in replication and survival of the parasite etc. Moreover, the biochemical analysis of *Plasmodium falciparum* transketolase (PfTk) shows least homology with its human host [15]. All these make it a potential target for treating malaria.

The preliminary aim of the non-oxidative arm of the PPP is to generate ribose-5-phosphate (R5P). But when two carbon groups are transferred from xylulose-5-phosphate to ribose-5-phosphate it generates glyceraldehyde-3-phosphate (G3P), fructose-3-phosphate (F6P) and sedoheptulose-7-phosphate. This transfer reaction is catalyzed by transketolase and as a co-factor it requires thiamine diphosphate (ThDP). Transketolase is also responsible for the production of erythrose-4-phosphate from F6P and G3P in the absence transaldolase which is another enzyme of the non-oxidative arm [16]. The R5P is used for the synthesis of nucleotides and nucleic acids. Therefore, the non-oxidative part of PPP is directly or indirectly responsible for generating more than 80 % of the parasite nucleic acid [17]. Moreover, Erythrose-4-phosphate is required as a key metabolite in the shikimate pathway. It produces chorismate which is an aromatic precursor. This can be further metabolized into other aromatic compounds such as folate. As shikimate pathway is present in *Plasmodium falciparum* and is absent in mammals, the enzymes of the pathway can be strongly considered as an effective drug target against malaria [18–21].

In the current study *Plasmodium falciparum* transketolase was subjected to extensive computational study to determine its chemical and structural properties along with its protein -protein interaction network. The study also predicted good quality model of Pftk using homology modeling techniques and subsequent computer aided active site prediction and docking simulation studies for the development of an effective drug against *Plasmodium falciparum* 3D7.

## Materials and methods

### Sequence retrieval

The amino acid sequences of transketolase [Accession XP_966097.1] of *P. falciparum* 3D7 were retrieved from the protein database of National Center for Biotechnology Information (NCBI). The protein is 672 amino acids long and used for further analysis in the current study.

### Primary structure prediction

ExPasy’s ProtParam tool [22] was utilized to calculate the physico-chemical characteristics of the protein. Theoretical isoelectric point (pI), molecular weight, total number of positive and negative residues, extinction coefficient [23], instability index [24], aliphatic index [25] and grand average hydropathicity (GRAVY) of the protein were calculated using the default parameters.

### Secondary structure analysis

Secondary structure was predicted by using the self-optimized prediction method with alignment (SOPMA). Protein’s secondary structural properties are including α helix, 3_10_ helix, Pi helix, Beta Bridge, Extended strand, Bend region, Beta turns, Random coil, Ambiguous states and other states [26].

### Disease causing region prediction

GlobPlot 2.3 was used to find out the disease causing regions of the protein. This web service looks for order/globularity or disorder tendency in the query protein based on a running sum of the propensity for an amino acid to be in ordered or disordered state by searching domain databases and known disorders in proteins [27].

### Template selection

To find out suitable template for the protein PSI (Position Specific Iterative) BLAST is performed against PDB database considering the default parameters except PSI-BLAST threshold to 0.0001. Total three iterations of PSI-BLAST were considered as the BLAST search results converged after three iterations [28]. The PDB structures of 1ITZ_A, 1AY0, 1TKA, 1TRK were selected as template structure.

### Template sequence alignment

Query sequence and the best template sequence according to identity parameter were aligned by Clustal Omega, the latest of Clustal family. Clustal omega algorithm takes input of an amino acid sequence then produces a pairwise alignment using k-tuple method followed by sequence clustering through mBed method and k-means clustering method. Final output of multiple sequence alignment is done by HHalign package, which aligns two profile hidden Markov models [29].

### Homology modeling

The model was generated using a comparative modeling program MODELLER9v13 [30] which generates a refined three dimensional homology model of a protein sequence based on a given sequence alignment and selected template. Homology modeling is able to produce high quality models provided that the query and template molecule are closely related. But model quality can decrease if sequence identity of target and template sequence falls below 20 % though it’s proven that protein structures are more conserved than their sequences [31]. The MODELLER generated five structures with 1ITZ_A, 1AY0, 1TKA, 1TRK as template structures from which the best one is selected on the basis of lowest discrete optimized protein energy (DOPE) score and highest GA341 score [32].

### Structure refinement

Modrefiner [33] is an algorithm for atomic-level, high-resolution protein structure refinement, which can start from C-alpha trace, main-chain model or full-atomic model. Modrefiner refine protein structures from Cα traces based on a two-step atomic-level energy minimization. The main-chain structures are first constructed from initial Cα traces and the side-chain rotamers are then refined together with the backbone atoms with the use of a composite physics and knowledge-based force field.

### Verification and validation of the structure

The accuracy and stereo chemical feature of the predicted model was calculated with PROCHECK [34] by Ramachandran Plot analysis [35] which was done through “Protein structure and model assessment tools” of SWISS-MODEL workspace. The best model was selected based on overall G-factor, number of residues in core, allowed, generously allowed and disallowed regions. Verify3D [36], ERRAT [37] and QMEAN [38] were used for additional analysis of the selected model. Finally, the protein was visualized by Swiss-PDB Viewer [39].

### Network interaction

STRING [40] was used to identify protein-protein interaction. STRING is a biological database which is used to construct Protein-protein interaction network for different known and predicted protein interactions. At present, string database covers up to 5,214,234 proteins from 1133 organisms [41]. RING (Residue Interaction Network Generator) was used to analyze residue-residue interaction of transketolase and generated network was visualized by Cytoscape 3.1.0 [42].

### Active site analysis

After modeling the three dimensional structure of transketolase, the probable binding sites of the protein was searched based on the structural association of template and the model construct with Computed Atlas of Surface Topography of proteins (CASTp) [43] server. CASTp was used to recognize and determine the binding sites, surface structural pockets, active sites, area, shape and volume of every pocket and internal cavities of proteins. It could be also used to calculate the number, boundary of mouth openings of every pocket, molecular reachable surface and area [44]. Active site analysis provides a significant insight of the docking simulation study.

### Docking simulation study

*In silico* docking simulation study, was carried out to recognize the inhibiting potential against Transketolase enzyme. Docking study was performed by Autodock vina [45]. Before starting the docking stimulation study, transketolase was modified by adding polar hydrogen. A grid box (Box size: 76 × 76 × 76 Å and box center: 11 × 90.5 × 57.5 for x, y, and z, respectively) was designed in which nine binding modes were generated for the most favorable bindings. The overall combined binding with Transketolase and 6′-Methyl-Thiamin Diphosphate was obtained by using PyMOL (The PyMOL Molecular Graphics System, Version 1.5.0.4, Schrödinger, LLC).

## Results

### Primary and Secondary structure analysis

ProtParam computes several parameters analysing the primary structure of the protein sequence. This parameters are the deciding functions of the proteins stability and function. The primary structure of a protein encodes motifs that are of functional importance, structure and function are correlated for any biological molecule. Secondary structural features of the protein are predicted by SOPMA algorithm. Both the results of primary and secondary structure analysis of the protein are presented in Table 1 and Table 2 respectively.

**Table 1:** Different physico-chemical properties of transketolase (*Plasmodium falciparum* 3D7)

Parameter	Value
Molecular weight	75815.2
Extinction coefficients	82460
Abs 0.1 % (=1 g/l) 1.088, assuming all pairs of Cys residues form cystines	
Ext. coefficient	81710
Abs 0.1 % (=1 g/l) 1.078, assuming all Cys residues are reduced	
Theoretical pI	6.50
Total number of negatively charged residues (Asp + Glu):	76
Total number of positively charged residues (Arg + Lys):	70
Instability index	38
Grand average of hydropathicity (GRAVY)	-0.402
Aliphatic index	82.89

**Table 2:** Secondary structure analysis through SOPMA of transketolase (*Plasmodium falciparum* 3D7)

Secondary Structure	Percentage
Alpha helix (Hh)	43.30 %
Extended strand (Ee) :	15.62 %
Beta turn (Tt) :	8.04 %
Random coil (Cc) :	33.04 %
3_10_ Helix	0.00 %
π helix	0.00 %
Isolated β-bridge	0.00 %
Bend	0.00 %

### Disease causing region prediction

**Fig. 1:**
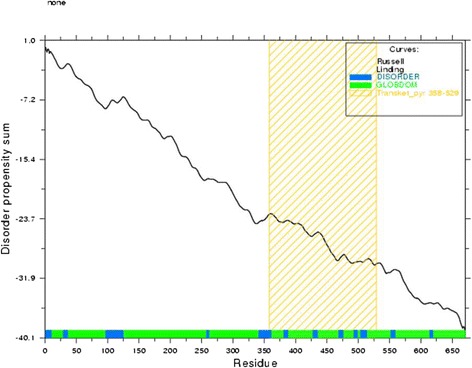
Globplot result shows the disease causing regions of transketolase

12 disorder regions were identified by GlobPlot. The result is shown in Fig. 1. The regions are from amino acid number 1-10, 29-36, 97-125, 258-262, 341-361, 381-388, 428-435, 469-476, 493-499, 504-514, 552-559 and 614-619.

### Allignment of target sequence

**Fig. 2:**
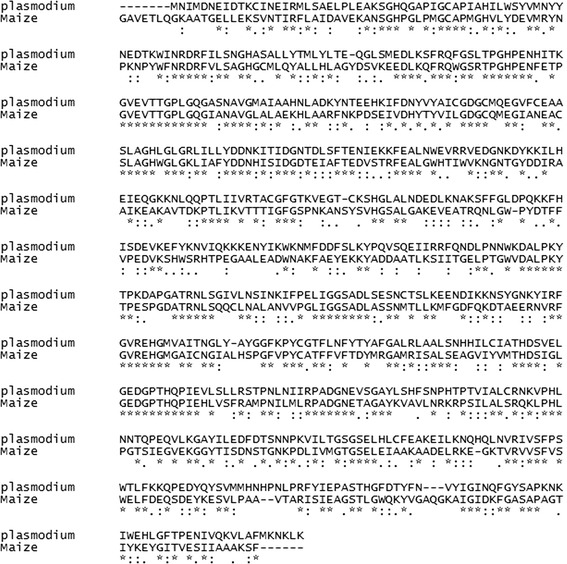
Sequence alignment of the template protein and the query protein sequences

Allignment between the target sequences and selected sequence was determined by clustal omega (Fig. 2). Clustal omega algorithm aligns sequences faster and more accurately. A good alignment of template sequences along with closely related template models are necessary for predicting a better quality model of the query protein through homology modelling.

### Model building

MODELLER 9.13 was used to determine the three dimensional (3D) model of the targeted protein. 3D protein structures provide valuable insights into the molecular basis of protein function. MODELLER generated result shows transketolase contains <90 % residues in favored region and 0.8 % of amino acids in the disallowed region.

### Refinement of the predicted model

**Fig. 3:**
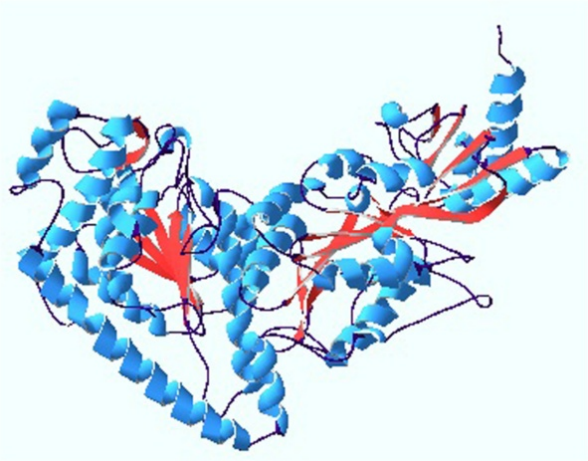
Refined model of Transketolase

MODELLER generated model was considered for further refinement through Modrefiner to gain a better quality structure. An increase of about 4 % residue in favored region is seen and other parameters acquired better acceptable value. The refined model is depicted in Fig. 3.

### Model verification and validation

**Fig. 4:**
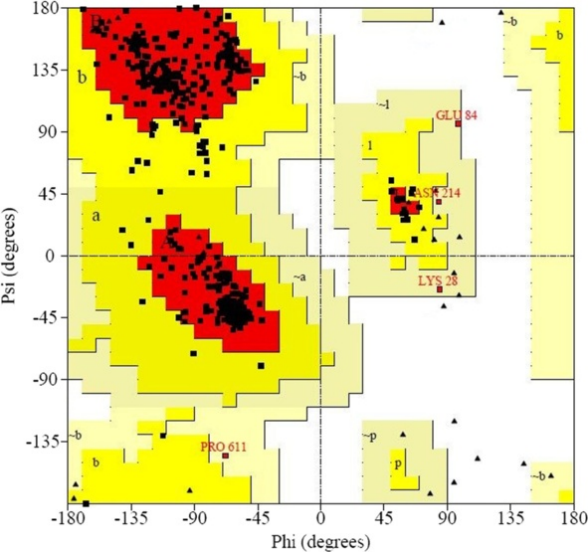
Ramachandran plot analysis of transketolase. Here, red region indicates favored region, yellow region for allowed and light yellow shows generously allowed region and white for disallowed region. Phi and Psi angels determine torsion angels

**Fig. 5:**
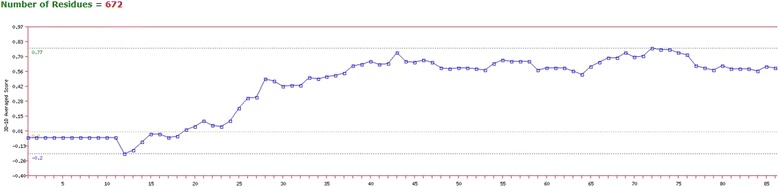
Verify 3D graph of transketolase (*P. falciparum* 3D7)

**Fig. 6:**
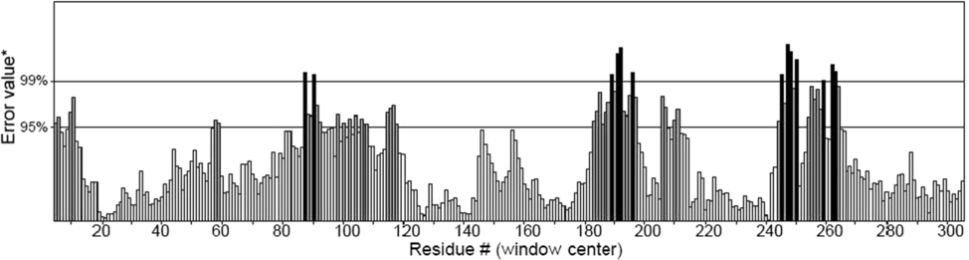
ERRAT generated result of transketolase where 95 % indicates rejection limit

**Fig. 7:**
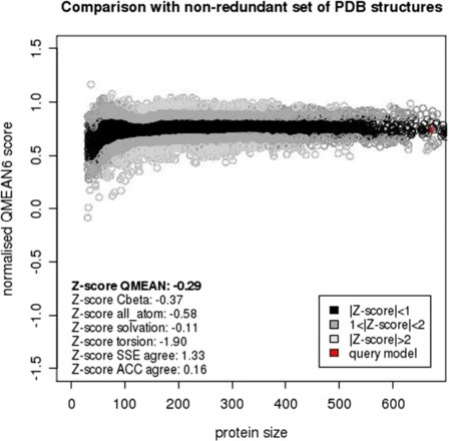
Graphical presentation of estimation of absolute quality of model transketolase (*P. falciparum* 3D7). Here the dark zone indicates that the model has a score <1. Models considered good are expected to position in the dark zone. The red marker shows a generated target model, which are considered to be a good model according to their position near or in the dark zone

Ramachandran plot was done by PROCHECK to measure the accuracy of protein model. The results were narrated in Table 3 and Fig. 4. The profile score above zero in the Verify3D graph correspond to the acceptable environment of the model, in Fig. 5. ERRAT; which verifies protein structure, generated result depicted in Fig. 6. QMEAN server was used for the verification of protein model which is shown in Fig. 7.

**Table 3:** Ramachandran plot of transketolase from *Plasmodium falciparum* 3D7

Ramachandran plot statistics	Transketolase	
	Residue	%
Residues in the most favored regions [A,B,L]	547	92.7
Residues in the additional allowed regions [a,b,l,p]	40	6.8
Residues in the generously allowed regions [a,b,l,p]	3	0.5
Residues in the disallowed regions [xx]	0	0.0
Number of non-glycine and non-proline residues	590	100.0
Number of end residues (excl. Gly and Pro)	2	
Number of glycine residues	49	
Number of proline residues	31	
Total number of residues	672

### Network generation

**Fig. 8:**
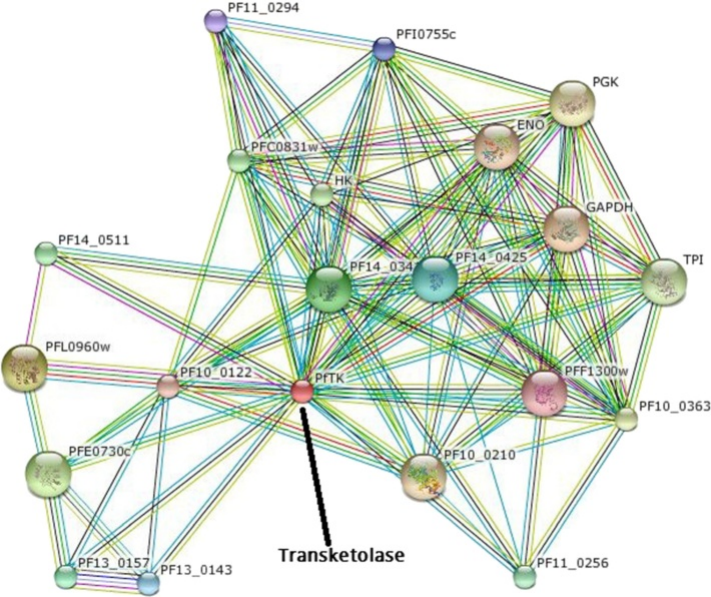
Protein-Protein Interaction network of transketolase (*Plasmodium falciparum* 3D7) detected through STRING

**Fig. 9:**
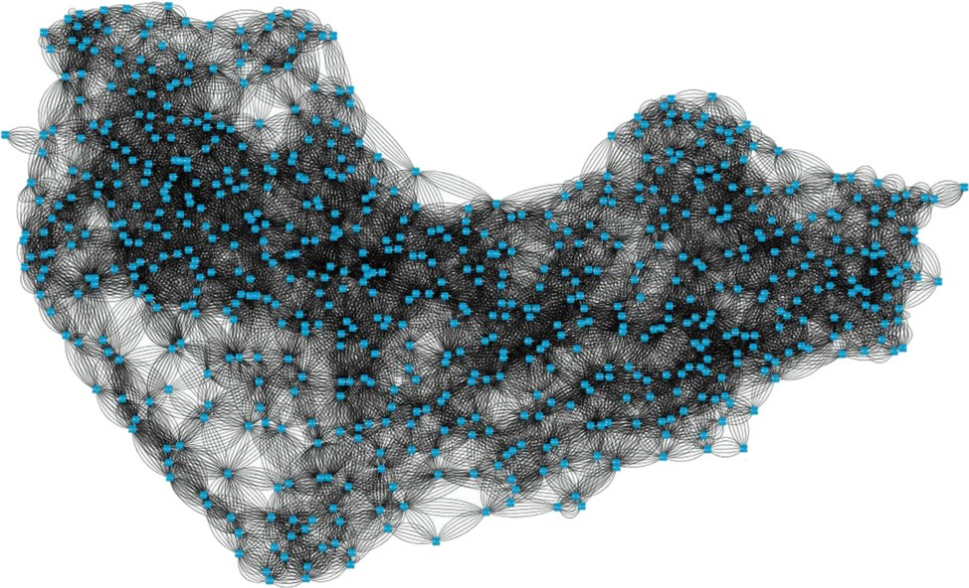
Residue interaction network generated by RING was visualized by Cytoscape. Here, nodes represent amino acids and edges represent interaction

The protein-protein interacting partners of Transketolase of *Plasmodium falciparum* 3D7 was determined by STRING (Fig. 8). Residue interaction network was depicted in Fig. 9.

### Active site prediction

**Fig. 10:**
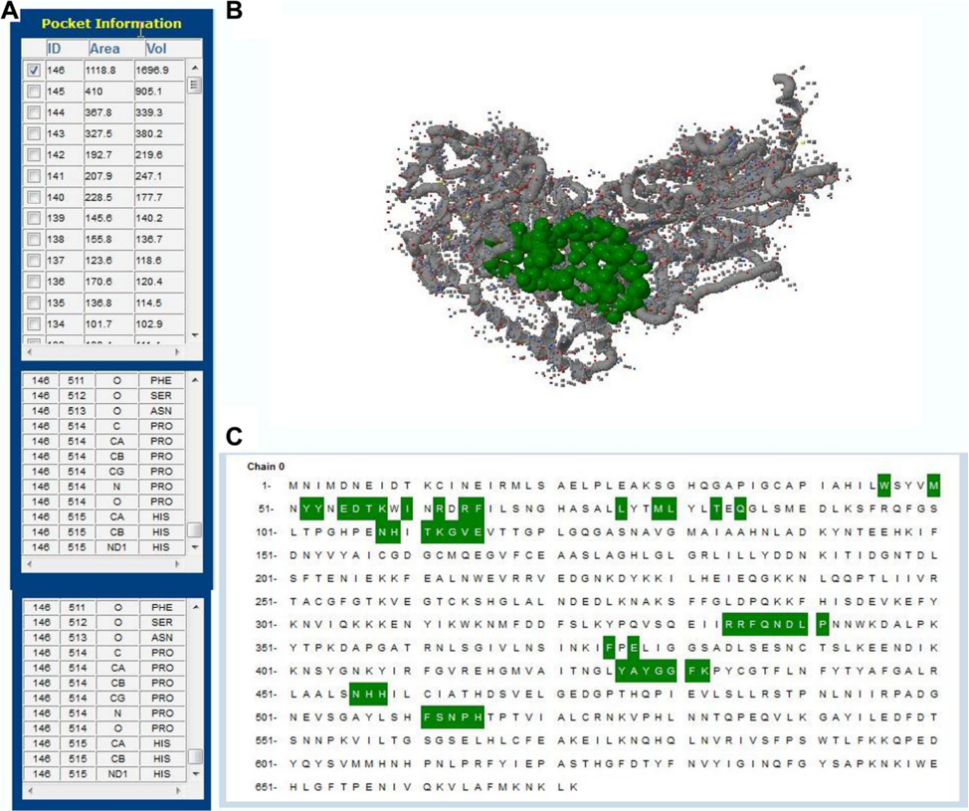
**a** The table of the area and the volume for different active sites of transketolase. **b** The Three Dimensional structure of the best active site. **c** Active site analysis by CASTp server. Green color illustrates the active site position from 46 to 515 with the beta-sheet in connecting them

The active site of transketolase was predicted by using CASTp server. The calculated result shows that the amino acid position 46-515 is predicted to be conserved with the active site. At this point, it is considered that the experimental binding sites of 6′-Methyl-Thiamin Diphosphate include some of the residues as stated above. Therefore in our study His 109, Asn 108 and His 515 are chosen as the more positive sites to dock the substrate. The number of pockets, their area and volume are graphically represented (Fig. 10).

### Docking results analysis

The exploration for the top ways is to fit ligand molecules into transketolase structure, using Autodock Vina resulted in docking files that included complete records of docking. The obtained log file is given in Table 4. The resemblance of docked structures was computed by calculating the root mean square deviation (RMSD) between the coordinates of the atoms and forming the clusters of the conformations based on the RMSD values. The lowest binding energy conformation in all cluster were considered as the most favorable docking pose. Binding energies that are reported signify the sum of the total intermolecular energy, total internal energy and torsional free energy minus the energy of the unbound system. The top nine ligands conformation were generated based on the energy value through Autodock Vina.

**Table 4:** Binding energies (kcal/mol) of the compounds along with their Root Mean Square Distance value obtained from Autodock Vina tool

Compound	1	2	3	4	5	6	7	8	9
6′-Methyl-Thiamin Diphosphate	-6.6	-6.4	-6.0	-5.4	-5.4	-5.4	-5.1	-5.1	-5.0
dist from best mode rmsd l.b.	0.000	3.252	2.378	3.123	4.875	2.724	5.149	25.545	26.623
dist from best mode rmsd u.b.	0.000	4.402	5.402	6.050	5.978	4.884	7.100	28.035	28.663

## Discussion

*Plasmodium falciparum* transketolase (pftk) is an attractive target site candidate for anti-malarial drug discovery. As the crystal structure of Pftk is unavailable, the homology modeling technique stands out as an excellent and powerful alternative to predict a reliable 3-D structure of the protein.

A physico-chemical analysis of the protein sequence was done by the Expasy server’s ProtParam tool. It revealed an instability index of 38.00, which denotes, this protein will be stable in-vitro because a value over 40 is considered unstable. The instability index is estimated from a statistical analysis of 12 unstable and 32 stable proteins where it was found that occurrence of certain dipeptides are significantly different among stable and unstable proteins. This protein was also predicted to have high aliphatic index; it is the total volume occupied by aliphatic side chains and higher value is considered a positive factor for increased thermo stability. Along with high extinction coefficient and negative GRAVY, the extents of other parameters imply the stability of the protein [46].

Results generated by secondary structure prediction tool SOPMA showed the enzyme is dominated by 43.3 % alpha helix and 33.04 % random coils along with 15.62 % extended strands and 8.04 % beta turns. The abundance of coiled region indicates higher conservation and stability of the model [47, 48].

High degree of flexibility in polypeptide chain and insufficiency of regular secondary structure is considered as disorder in protein [49]. Disordered regions might contain functional sites or linear motifs and many proteins are intrinsically found disordered *in vivo*. In Fig. 1 the blue colored sections on the X-axis are disordered regions and green colored regions are globular or ordered domains. Disordered regions are important because many intrinsically disordered proteins exist as unstructured and become structured when bound to another molecule [50, 51].

The 3D model of the Pftk derived from Modeller v.9 had 89.8 % of all its residues in the favorable region, 9.0 % and 0.3 % in allowed and generously allowed region. Only 0.8 % of the residues was in the disallowed region in the Ramachandran plot analysis where the amino acid residues of a peptide are plotted in favorable, allowed and disallowed regions according to their torsion angles phi (φ) and psi (ψ). Though homology modeling algorithm is one of the most robust modeling tools in bioinformatics, this often contain significant local distortions, including steric clashes, unphysical phi/psi angles and irregular H-hydrogen bonding networks, which make the structure models less useful for high-resolution functional analysis. Refining the modeled structures could be a solution of this problem [52]. Refinement through Modrefiner has depicted 92.7 % of its entire residue in the most favored regions, 6.8 % in the additional allowed regions, 0.5 % in the generously allowed regions and 0.0 % in disallowed regions. The statistics of the refined model showed that majority of the residues fall in the favorable core region including all non-glycine and non-proline residues, in the Ramachandran plot, it ensures good stereo-chemical quality of the model.

From the refined structures the best structure has been selected using structure validation tools; namely PROCHECK, Verify 3D and ERRAT. The highest scoring structure was picked as the final structure. VERIFY 3D uses the 3D profile of a structure to determine its correctness by matching it with its own amino acid sequence. A high score match is expected between the three dimensional profile of a structure and its own sequence. This compatibility score of an atomic model (3D) with its sequence (1D) ranges from -1 (bad) to +1 (good), so, score 0.77 in verify 3D determines good environmental profile of the structure [53]. ERRAT, the structure verification algorithm interpreted the overall quality of the model with the resulting score 78.313; this score denotes the percentage of the protein that falls below the rejection limit of 95 % [37].

The QMEAN scoring function estimates the geometrical aspects of a protein structure by a composite function of six different structural descriptors; a torsion angle potential over three consecutive amino acids to analyze local geometry, long range interactions assessed by a secondary structure-specific distance-dependent pairwise residue-level potential, a solvation potential describing the the burial status of the residues and two agreement term determining the agreement of predicted and calculated secondary structure and solvent accessibility [38, 54]. The Z-scores of the QMEAN terms of the protein model are -0.37, -0.58, -0.11, -1.90, 1.33, 0.16 for C_β interaction energy, salvation energy, torsion angle energy, secondary structure, and solvent accessibility respectively. These scores indicate that the predicted protein model can be considered as a good model. Moreover, to estimate the absolute quality of the model the QMEAN server [55] relates the query model with a representative set of high resolution X-ray structures of similar size and the resulting QMEAN Z-score is an extent of “degree of nativeness” of the given structure [56]. The average z-score of high resolution models is ‘0’. The QMEAN z-score for the query model is -0.29, which is lower than the standard deviation ‘1’ from the mean value ‘0’ of good models, so, this result shows that the predicted model is of comparable quality to the high resolution models. In addition the range of predicted global model reliability is 0 to 1 according to Verify 3D. Hence, *Plasmodium falciparum* transketolase with a global model reliability score 0.74 has all the potentials of a good quality model [57–59].

Protein-protein interaction (PPI) networks generation have become crucial tool of modern biomedical research for the understanding of intricate molecular mechanisms and for the recognition of novel modulators of disease progressions. To study varieties of human diseases as well as their signaling pathways, protein interactions give an immense effect [60–62]. PPI of Transketolase generated through STRING is presented in (Fig. 8). STRING forecasts a confidence score, 3D structures of protein and Protein domains. STRING utilizes references from UniProt (Universal Protein) resource and predicts functions of different interacting protein. PPI network demonstrates that transketolase interacts with twenty other proteins in a high confidence score among which GAPDH (Glyceraldehyde 3-phosphate dehydrogenase); an exosomal protein that functions in some crucial pathways like glycolysis/gluconeogenesis and amino acid biosynthesis. D-ribulose-5-phosphate 3-epimerase, is the enzyme that converts D-ribulose 5-phosphate into D-xylulose 5-phosphate in Calvin’s reductive pentose phosphate cycle [63]. ENO stands for enolase, also known as 2-phospho-D-glycerate hydro-lyase which is a metalloenzyme responsible for the catalyting of the conversion of 2-phosphoglycerate (2-PG) to phosphoenolpyruvate (PEP).

Residue interaction networks (RINs) have been used to describe the protein three-dimensional structure as a graph where nodes and edges represent residues and physico-chemical interactions respectively. To analyze residue-residue interaction, protein stability and folding, allosteric communication, enzyme catalysis or mutation effect prediction RING is being used. RING uses standard programs to create network interaction that is visualized through Cytoscape [64–67]. Cytoscape is an open source software package for visualizing, modeling and analyzing molecular and genetic interaction networks. A higher bonding interaction indicates higher probability of protein functioning site [68–70]. Residue-residue interaction network of transketolase indicates the probable active site of the crucial protein of *plasmodium falciparum* [71].

The active site of transketolase was predicted by CASTp server as shown in Fig. 10. In our present study, we reported the surpass active site area of the enzyme in addition to the number of amino acids occupied in it. The preeminent active site is found with 1118.8 areas and a volume of 1696.9 amino acids.

**Fig. 11:**
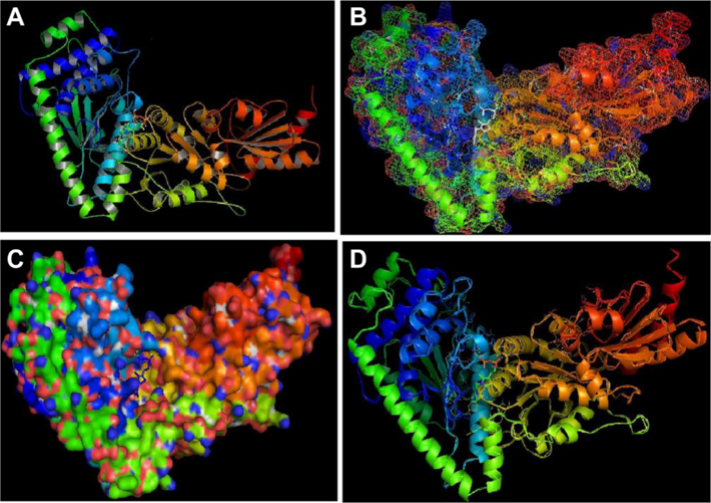
The overall binding between the transketolase and 6′-Methyl-Thiamin Diphosphate. **a** Biological assembly of transketolase and 6′-Methyl-Thiamin Diphosphate, **b** Mesh structure of transketolase and 6′-Methyl-Thiamin Diphosphate, **c** Surface structure of transketolase and 6′-Methyl-Thiamin Diphosphate, **d** Cartoon structure of transketolase and 6′-Methyl-Thiamin Diphosphate

**Fig. 12:**
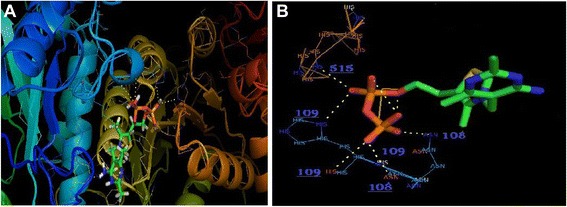
Graphical Representation of docking study between 6′-Methyl-Thiamin Diphosphate and Transketolase (yellow dashed-lines indicate hydrogen bonds). **a** Visualization of 6′-Methyl-Thiamin Diphosphate-Transketolase interaction **b** Hydrogen Bond detection through PyMOL

The complete profile of the studies by AutoDock Vina, is represented in Table 5. For the most favorable binding 6′-Methyl-Thiamin Diphosphate, estimated free energy of molecular binding was of −6.6 kcal/mol. The overall binding energies as well as RMSD (Å) of 6′-Methyl-Thiamin Diphosphate based on their rank are tabulated in Table 4. Overall binding of transketolase and 6′-Methyl-Thiamin Diphosphate is represented in Fig. 11. It has been found that 6′-Methyl-Thiamin Diphosphate formed 5 Hydrogen bonds with the transketolase (Fig. 12). The Amino acid residues conscientious for the binding interactions of the 6′-Methyl-Thiamin Diphosphate (Fig. 11b) with the enzyme are His 109, His 515, Asn 108**.** The description of 6′-Methyl-Thiamin Diphosphate is given in Table 6. After analyzing the results, in case of our selected ligand it is clearly concluded that this has a crucial role in ligand binding affinity.

**Table 5:** Comparative docking study of the ligand to the target

Ligand	Protein	No. of H bonds	Interacting residues
6′-Methyl-Thiamin Diphosphate	Transketolase	5	His 109, Asn 108, His 515,

**Table 6:** Description of Ligand molecule

Name	6′-Methyl-Thiamin Diphosphate	Chemical structure
Identifiers	[2-[3-[(4-amino-2,6-dimethyl-pyrimidin-5-yl)methyl]-4-methyl-1,3-thiazol-3-ium-5-yl]ethoxy-hydroxy-phosphoryl] hydrogen phosphate	
Formula	C_13_ H_20_ N_4_ O_7_ P_2_ S	
Molecular Weight	438.33 g/mol	
Type	non-polymer	

## Conclusion

By analyzing different structural and physiological parameters of *P. falciparum* 3D7, in this study we predicted the 3D structure of PfTk. Evidences have shown that, PfTk (transketolase) can be considered as a remarkable drug target for its role in the regulation of non-oxidative arm of the PPP and for the least homology with its human host. The need of a proper vaccine against malaria has never been more serious as malaria increasingly claiming life in this 21^st^ century. This study is aimed to aid the hunt for the proper target site in the quest for a sole solution to defend malaria. The structural information of our given model will pave the way for further laboratory experiments to design potential anti-malarial drug in near future.

## Abbreviations

Pftk: *Plasmodium falciparum* transketolase
GRAVY: Grand average hydropathicity
SOPMA: Self-optimized prediction method with alignment
PDB: Protein data bank
STRING: Search tool for the retrieval of interacting genes/proteins
RING: Residue interaction network generator
CASTp: Computed atlas of surface topography of proteins
RMSD: Root mean square deviation
PPI: Protein-protein interaction
